# Organ‐specific transcriptional and metabolic adaptations of potato plants to limited phosphate availability prior and after tuberization

**DOI:** 10.1111/tpj.70445

**Published:** 2025-09-05

**Authors:** Maryam Nasr Esfahani, Lisa Koch, Jörg Hofmann, Sophia Sonnewald, Uwe Sonnewald

**Affiliations:** ^1^ Department of Biology, Chair of Biochemistry Friedrich‐Alexander‐University Erlangen‐Nuremberg Erlangen Germany

**Keywords:** phosphorus‐availability fluctuations, *Solanum tuberosum*, phosphate starvation‐responsive genes, phosphorus use efficiency

## Abstract

While plants adapt to fluctuating phosphorus (P) availability in soils by enhancing phosphate acquisition or optimizing internal P‐utilization, the spatiotemporal dynamics of these responses, particularly in crops, remain poorly understood. This study systematically investigated how and when potato organs respond to fluctuating P availability across different developmental stages using transcriptomic, metabolomic, and physiological analyses of leaves, roots, and tubers. Transcriptomic data revealed dynamic, organ‐ and stage‐specific responses to P‐deficiency, with the highest number of differentially expressed genes in leaves before tuberization and in roots during tuberization. P‐deficiency led to a marked accumulation of proline in tubers and nitrogen‐rich amino acids, particularly glutamine and asparagine, in roots and leaves. Carbohydrate metabolism exhibited severity‐ and time‐dependent changes: severe P‐deficiency triggered earlier, stronger, but transient carbohydrate accumulation, whereas medium P‐deficiency led to a gradual and sustained increase in leaves and roots. Hexose phosphates and organic acids accumulated in roots under P‐stress, especially severe P‐stress, during early vegetative growth, followed by a marked reduction during tuberization. During tuber filling, severe P‐deficiency reduced sucrose and starch in roots, decreased leaf starch but increased leaf sucrose, likely due to impaired translocation, and a decrease in tuber sucrose alongside increased starch due to reduced degradation. Under medium P‐deficiency, sucrose and starch remained stable in leaves and tubers but declined in roots, reflecting a moderate shift in carbon allocation that maintained tuber development at the expense of root metabolism. These findings highlight the spatiotemporal regulation of metabolic and molecular responses to P‐deficiency in potato and provide insights for improving nutrient use efficiency and stress resilience in crops.

## Significance STATMENT

This study reveals that potato plants exhibit distinct, stage‐ and organ‐specific transcriptional and metabolic responses to phosphorus deficiency, with major shifts in amino acid and carbohydrate metabolism. These findings highlight how plants dynamically reallocate carbon and nitrogen resources to optimize growth and survival under fluctuating P availability, offering new targets for improving nutrient use efficiency.

## INTRODUCTION

Phosphorus (P) plays a crucial role in many cellular processes, including photosynthesis, respiration, and the biosynthesis of ATP, nucleic acids, and membranes. As a key macronutrient, P frequently limits productivity in both natural and agricultural ecosystems, notably affecting crop production in low‐input systems worldwide (Lambers, 2022). Despite the abundance of P in the lithosphere, its plant‐utilizable form, inorganic orthophosphate, including HPO_4_^2−^ and H_2_PO_4_^−^, is insoluble and diffuses slowly in soils, resulting in P‐deficiency in agricultural lands and ecosystems (Paz‐Ares et al., 2022). Therefore, applying P fertilizers is a rapid and conventional approach to rectify P‐deficiency. However, the root system can only take up less than 30% of the applied P. A notable proportion of applied P is rapidly immobilized by binding with magnesium and calcium cations in calcareous soils or with iron and aluminum ions in acidic soils, and consequently is unavailable for uptake or is lost in water bodies, causing eutrophication, which is a serious environmental problem (López‐Arredondo et al., 2014). Additionally, at the current rate of global phosphate rock consumption for P fertilizer production, the reserves of nonrenewable phosphate rock would be depleted within 100 years or less. Since P fertilizers have no replacement or alternative, we will run into a food production crisis in less than a century due to the lack of sufficient P fertilizers (Herrera‐Estrella & López‐Arredondo, 2016). Hence, there is a need for innovative strategies to engineer P‐efficient crops, enabling them to utilize limited P resources more effectively and to ensure the sustainability of agriculture. This requires a comprehensive understanding of how P regulates diverse metabolic and molecular processes, forming the foundation for developing crop varieties with higher P‐efficiency, thereby contributing to the efficient management of P resources. Most studies on P‐deficiency have focused on plant responses during a single developmental stage, typically the vegetative stage, as seen in research in Arabidopsis (Lan et al., 2012; Misson et al., 2005; Müller et al., 2007), rice (Prathap et al., 2023), maize (Li et al., 2008), tomato (Satheesh et al., 2022), and barley (Nadira et al., 2016). However, the adaptive strategies of plants to P‐deficiency are intricately influenced by the extent of P scarcity, specific plant organs, and the developmental stage. Despite this, comprehensive analyses integrating metabolic and molecular responses across different organs, developmental stages, and levels of P‐deficiency in crops remain scarce, highlighting a critical gap in our understanding of when and how various plant organs adjust to fluctuating P‐availability.

Potato (*Solanum tuberosum*), the fourth most important food crop worldwide, produces tubers rich in carbohydrates, essential minerals, vitamins, and amino acids (AAs) as an excellent staple food in human diets. In addition, potato tubers are used as animal feed, in industrial applications, and as a food thickener, making it a highly versatile and valuable crop in an agricultural system (Zaheer & Akhtar, 2016). P‐availability significantly influences potato yield and tuber quality (Koch et al., 2020; Naumann et al., 2020). When all other nutrients are in sufficient quantities, potato plants exhibit a relatively higher P demand than other crops (Koch et al., 2020). For instance, the demand for P is approximately four times greater than the requirement for cereals needed to attain 95% of their potential yield (Nawara et al., 2017). The high P demand for potato can be attributed to its shallow root system and inefficient P‐uptake at low soil P concentrations (Dechassa et al., 2003), making P availability a critical limiting factor in potato production. The inherent sensitivity of the potato to low P‐availability (Hopkins & Hansen, 2019) presents a challenge in maintaining both high yield and quality through fertilization. Therefore, a deeper understanding of potato responses to changing P‐availability over time is essential for developing high P‐efficiency potato cultivars.

Thus, this study aimed to characterize potato plants' organ‐specific responses to variations in P‐supply at distinct developmental stages. We studied the integrative reactions of metabolism and global gene expression to severe P‐deficiency (0.05 mM P), medium P‐deficiency (0.5 mM P), and sufficient P‐availability (5 mM P) in different organs (leaf, root, and tuber) and developmental stages, namely, pre‐tuberization (1‐, 2‐, and 3‐week‐old plants), tuberization (6‐week‐old plants), and tuber filling (11‐week‐old plants). By examining these critical developmental stages, we obtained a more comprehensive understanding of potato plants' adaptive molecular and metabolic strategies to cope with P deficiency throughout their growth cycle, which could contribute to developing more P‐efficient potato cultivars and increasing global food security.

## RESULTS AND DISCUSSION

### Impact of fluctuations of available‐P on internal free phosphate dynamics and tuber yield in potato plants

**Figure 1:**
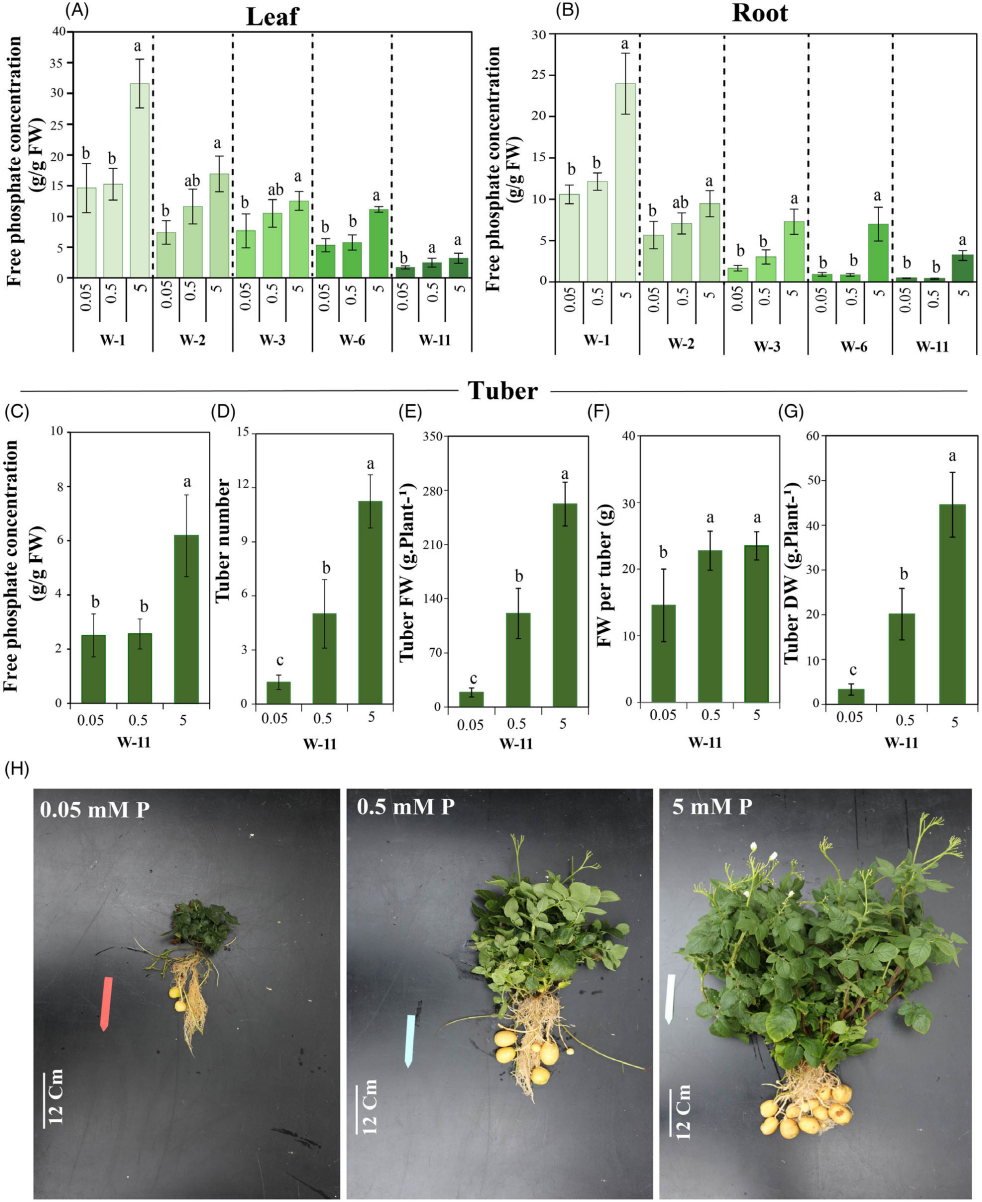
The effect of different P levels (0.05, 0.5, and 5 mM KH₂PO₄) on free phosphate concentration in the leaves (A), roots (B), and tubers (C); tuber number (D), tuber fresh weight (E), fresh weight per tuber (F), tuber dry weight (G) and representative images of 11‐week‐old potato plants grown under varying P‐availability conditions (H). Statistical analysis of data was performed at each developmental stage separately. Values represent means±SD of five biological replicates. Different letters indicate statistically significant differences based on Duncan's multiple range test (*P*≤0.05). DW, dry weight; FW, fresh weight; W, week.

Internal free phosphate levels in various organs were determined throughout development in potato plants grown for 11 weeks under severe P‐deficiency, medium P‐deficiency, and sufficient P‐availability. Plants sensed P‐deficiency primarily through decreased internal free phosphate levels, which trigger P‐starvation responses (Zhang, Liao, & Lucas, 2014). Internal free phosphate content in leaves and roots was measured at five time points to monitor its dynamic changes (Figure 1A,B). Under sufficient P‐availability, internal free phosphate levels in both roots and leaves remained consistently high during the pre‐tuberization (weeks 1, 2 and 3), tuberization (week 6), and tuber‐filling stages (week 11). This indicates that under adequate P‐supply, P‐uptake is sufficient to meet plant metabolic demands. In contrast, plants grown under severe and medium P‐deficiency exhibited a significant reduction in free phosphate concentrations in leaves, roots, and tubers (Figure 1A,B), reflecting limited external P availability and the plant's inability to replenish P consumed during cellular processes. Across all treatments, the marked decline in free phosphate levels in roots and leaves at the tuber‐filling stage (week 11) (Figure 1A,B) suggests that a significant portion of phosphate may have been allocated to the developing tubers. Medium and severe P‐deficiency decreased tuber free phosphate level and average tuber yields, both in number and weight (Figure 1C–H), aligning with previous studies that highlight the critical role of P‐availability in promoting overall plant growth and tuber formation (Koch et al., 2020; Sandaña & Kalazich, 2015).

### Organ‐ and developmental stage‐specific transcriptomic responses revealed distinct potato adaptation strategies to severe and medium P deficiency

To assess the impact of varying P‐availability on gene expression, RNA was extracted from the leaves and roots of plants grown under different P regimes at 3 to 6 weeks, followed by transcriptome analysis and the identification of differentially expressed genes (DEGs) compared to sufficient P availability.

**Figure 2:**
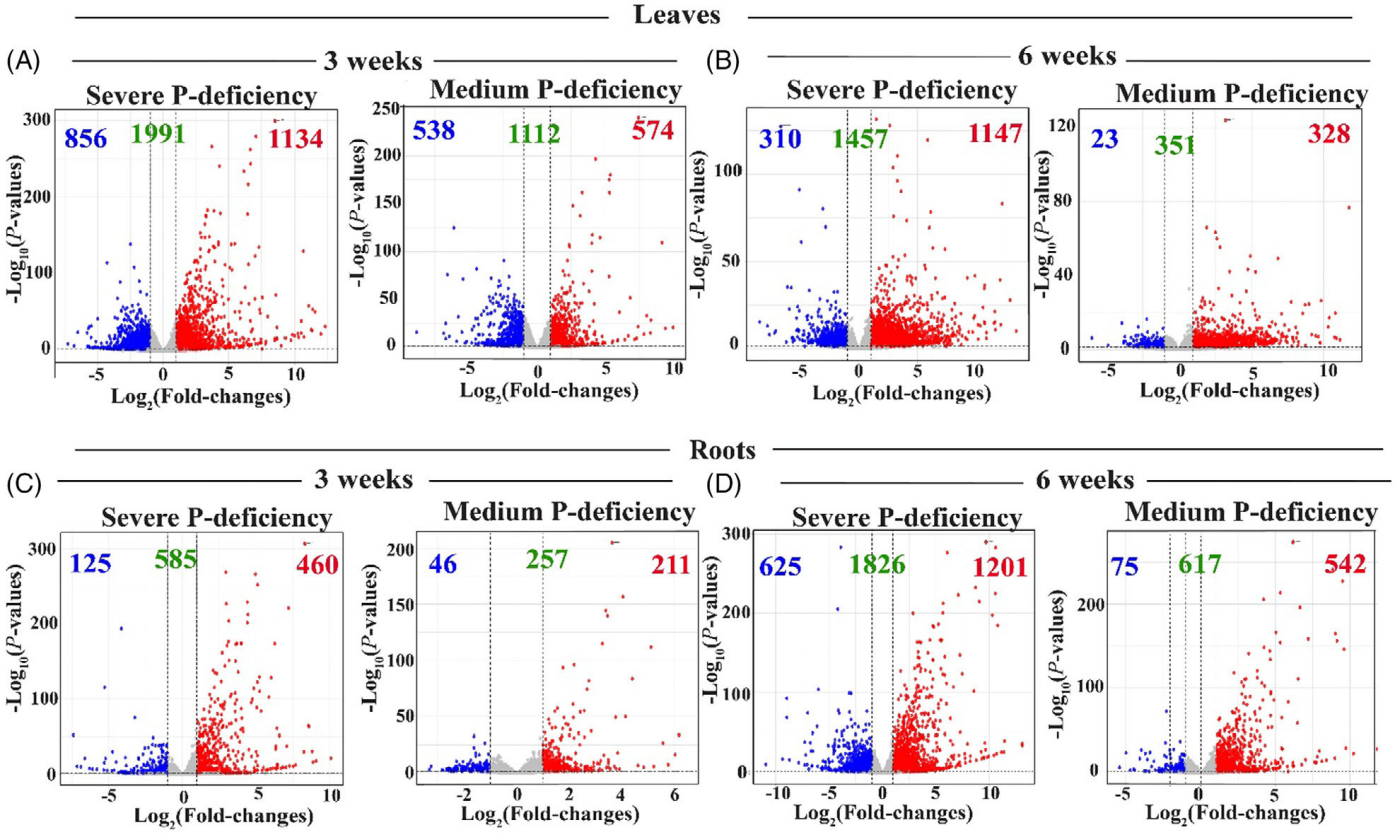
Volcano plots of significantly upregulated [log_2_ (fold changes) ≥1; *P*‐values ≤0.05] and downregulated [log_2_ (fold changes) ≤−1; *P*‐values ≤0.05] genes for two comparisons of ‘Severe P‐deficiency/Sufficient P‐availability’ and ‘Medium P‐deficiency/Sufficient P‐availability’ in the leaves (A, B) and roots (C, D) of potato plants grown under severe P‐deficiency (0.05 mM KH_2_PO_4_), medium P‐deficiency (0.5 mM KH_2_PO_4_), or sufficient P‐availability (5 mM KH_2_PO_4_) for 3 and 6 weeks. Red, blue, and green numbers indicate the number of upregulated, downregulated, and total differentially expressed genes, respectively.

Comparison of DEGs in roots and leaves under severe and medium P deficiency at week 3 (pre‐tuberization) and week 6 (tuberization) revealed distinct temporal and organ‐specific responses. At week 3, a higher number of DEGs was detected in the leaves than in the roots under both severe (1991 vs. 585) and medium (1112 vs. 257) P deficiency (Figure 2A,B; Tables S1–S4). In contrast, by week 6, the number of DEGs was greater in the roots than in leaves for both severe (1826 vs. 1457) and medium (617 vs. 351) P deficiency (Figure 2C,D; Tables S1‐S4). These findings highlight significant differences in the kinetics of transcriptional responses to varying P‐supply between organs, potentially reflecting distinct adaptive strategies employed by roots and leaves at different developmental stages. Principal component analysis (PCA) was applied to identify the main sources of transcriptional variations in potato plants under severe P‐deficiency, medium P‐deficiency, and sufficient P availability during the pre‐tuberization and tuberization stages (Figure S1). PC1 captured 71% of the variation, primarily separating samples by organ type, while PC2 accounted for 11%, distinguishing samples by developmental stage. This confirmed distinct adaptive strategies in roots and leaves across developmental stages in response to varying P levels (Figure S1a). Organ‐specific PCA further revealed that PC1 separated samples by developmental stage and PC2 by P treatment, indicating that gene expression variability within each organ was largely driven by the developmental stage (Figure S1b,c). Additionally, gene expression differences due to P treatments were more pronounced during tuberization than in the pre‐tuberization stage, as seen by stronger separation of the samples (Figure S1a–c).

Given that plant adaptive responses to increase efficient acquisition and utilization of phosphate, and consequently to cope with P starvation, are mainly regulated at the transcriptional level (Franco‐Zorrilla et al., 2004; Wang et al., 2018), the RNA‐seq data were investigated for genes encoding transcriptional regulators. In both severe and medium P‐deficiency, the number of up‐ or downregulated genes associated with the transcriptional regulation of *phosphate starvation‐induced* (*PSI*) genes in leaves was higher during the pre‐tuberization compared to the tuberization stage (139 vs. 110 for severe P‐deficiency and 88 vs. 11 for medium P‐deficiency) (Table S5a–d), while in roots, their expression was more pronounced during tuberization compared to the pre‐tuberization phase (156 vs. 37 for severe P‐deficiency and 44 vs. 25 for medium P‐deficiency) (Table S5e–h). Moreover, in both organs, the number of up‐ or down‐regulated genes encoding transcriptional regulators was higher in response to severe P‐deficiency than medium P‐deficiency (248 vs. 100 for leaves and 193 vs. 69 for roots) (Table S5a–h). Interestingly, few differentially expressed transcription factor (TF) genes were common between root and leaf organs, or across developmental stages, indicating distinct sets of TFs regulate potato responses to fluctuations of available P at pre‐tuberization and tuberization (Figure S2). For instance, in leaves under severe or medium P‐deficiency, *STARVATION RESPONSE REGULATOR 1 (StPHR1)/MYB‐CC* (*Soltu.DM.11G021700*) was upregulated during the vegetative stage, while *StMYB62* (*Soltu.DM.05G003270*), *StWRKY75* (*Soltu.DM.05G012130*), *StWRKY6* (*Soltu.DM.02G004200*, *Soltu.DM.02G020430*), and *StWRKY45* (*Soltu.DM.02G034320*) were upregulated during tuberization (Table S5a–d). In roots, *StPHR1/MYB‐CC* and *StMYB62* were induced under severe P‐deficiency at both developmental stages, whereas *StMYB62* and *StWRKY45* were upregulated under medium P‐deficiency specifically during tuberization (Table S5e–h). MYB62 (Devaiah et al., 2009), WRKY75 (Devaiah et al., 2007), WRKY6 (Chen et al., 2009) and WRKY45 (Wang, Xu, et al., 2014) are key TFs that regulate P starvation‐responsive genes, each modulating distinct aspects of the plant's adaptive response to P‐deficiency.

### Phosphate sensing and signaling in response to P fluctuations during pre‐tuberization and tuberization stages

Phosphate sensing and signaling are crucial for reprogramming the transcriptome and coordinating appropriate adaptive responses to fluctuations in P availability. SYG1/Pho81/XPR1 (SPX) domain‐containing proteins have emerged as key intracellular sensors in the P‐starvation signaling pathway (Duan et al., 2008; Secco et al., 2012; Wang, Ruan, et al., 2014). SPX domain‐containing proteins modulate the activity of PHR1/MYB‐CC TF, the principal regulator of *PSI* genes, in a phosphate‐dependent manner (Puga et al., 2014; Wang, Ruan, et al., 2014). In low‐P availability, transcription levels of *SPX1* and *SPX2* increase while the interaction affinity between SPX1/SPX2 and HPR1 decreases (Puga et al., 2014; Wang, Ruan, et al., 2014). As a result, HPR1 interacts with its targets (*PSI* genes), ultimately triggering their transcriptional induction. SPX3 is strongly induced by P starvation (Li et al., 2016; Wang, Hu, et al., 2009) and plays a positive role in plant adaptation to P starvation (Duan et al., 2008). Our results showed a significant increase in the transcripts of *StSPX1* (*Soltu.DM.08G011500*), *StSPX2* (*Soltu.DM.12G026570*), and *StSPX3* (*Soltu.DM.01G031540*) in both leaves and roots throughout the pre‐tuberization and tuberization stages under both severe and medium P‐deficiency, with the most pronounced expression levels observed for *StSPX3* (Tables S2 and S4). SPX4 has been identified as a negative regulator of some *PSI* genes (Osorio et al., 2019). For example, He et al. (2021) suggested that SPX4 binds to PRODUCTION OF ANTHOCYANIN PIGMENTS 1 (PAP1), a master MYB regulator of anthocyanin biosynthesis (Tohge et al., 2005), thereby inhibiting PAP1 binding to the promoter of genes encoding dihydroflavonol 4‐reductase (DFR), the rate‐limiting enzyme of anthocyanin biosynthesis (Saito et al., 2013). Phosphate starvation weakens the SPX4–PAP1 interaction, enabling PAP1 to bind the DFR promoter and activate anthocyanin biosynthesis, a metabolic marker of P‐deficiency (Steyn et al., 2002; Watanabe et al., 2013). Our findings revealed an upregulation of *StDFR* gene expression (Figure S3a), accompanied by increased anthocyanin accumulation in leaves exposed to severe P‐deficiency at both developmental stages (Figure S3b). Interestingly, this upregulation occurred despite the increased expression of *StSPX4* genes (*Soltu.DM.02G009540* and *Soltu.DM.02G031520*), indicating a potential weakening of the interaction PAP1–SPX4 under P‐starvation conditions (He et al., 2021). Severe P‐deficiency‐induced accumulation of anthocyanins in the leaves serves as a protective mechanism for chloroplasts from photo‐inhibitory damage in response to P‐deficiency (Pietrini et al., 2002).

### Transcriptional reprogramming in response to P fluctuations occurred via genes related to transport, lipid remodeling, phosphate recycling, and remobilization processes

#### Transporter‐related transcripts

**Figure 3:**
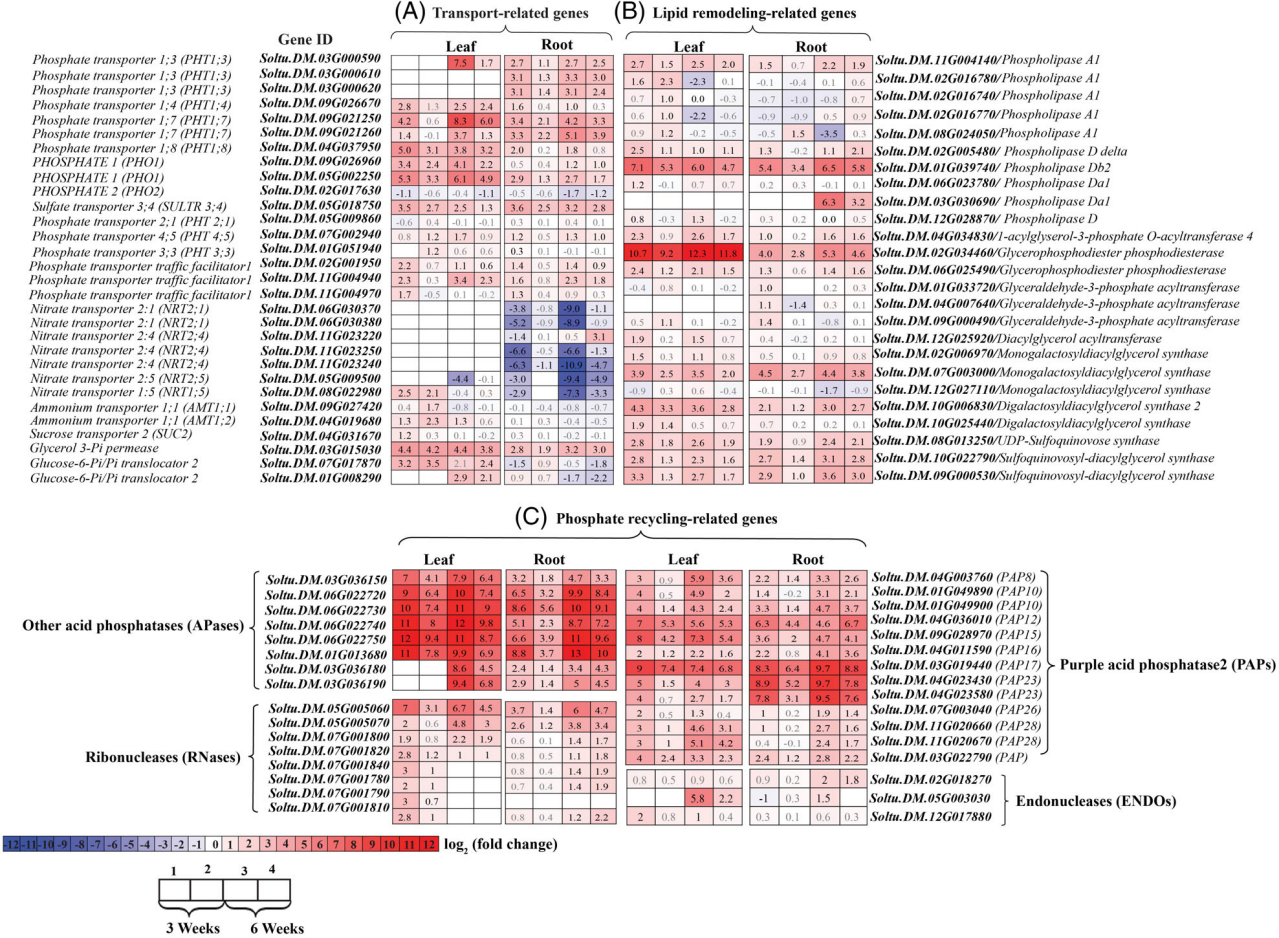
Changes in the expression of various transporter‐related genes (A) and genes encoding enzymes involved in membrane lipid remodeling (B) and phosphate cycling (C) in roots and leaves of potato plants grown under severe P‐deficiency (0.05 mM KH_2_PO_4_), medium P‐deficiency (0.5 mM KH_2_PO_4_), or sufficient P‐availability (5 mM KH_2_PO_4_) for 3 and 6 weeks. Changes in expression levels of genes are shown by intensities of colors expressed in log_2_ fold change, scales with saturation at twelve‐fold. Gray‐colored numbers indicate non‐significant changes. 1, ‘severe P‐deficiency versus sufficient P‐availability’; 2, ‘medium P‐deficiency versus sufficient P‐availability’; 3, ‘severe P‐deficiency versus sufficient P‐availability’; 4, ‘medium P‐deficiency versus sufficient P‐availability’.

Modifications in the abundance of phosphate transporters (PHTs)‐related transcripts are crucial strategies for adapting to fluctuations in P‐supply (Chien et al., 2022; Yang et al., 2024). Significant upregulation was observed for genes linked to phosphate acquisition from the soil (*PHT1;3* and *PHT1;4*) (Młodzińska & Zboińska, 2016), phosphate root‐to‐shoot translocation (*PHT1;8* and *PHO1*) (Hamburger et al., 2002; Lapis‐Gaza et al., 2014), and phosphate remobilization (*PHT1;7*, *PHT1;8* and *PHO1*) (Lapis‐Gaza et al., 2014), with greater increases under severe P‐deficiency than medium P‐deficiency during both pre‐tuberization and tuberization stages (Figure 3A). Interestingly, the peak expression of PHT genes under severe P‐deficiency occurred earlier (week 3), whereas under medium P‐deficiency, maximum expression was delayed to week 6, suggesting that the intensity of P‐stress influences the timing of transcriptional activation, with severe P‐deficiency triggering a more rapid response (Figure 3A). An increased expression of *PHO1*, *PHT1;7*, and *PHT1;8* genes, involved in phosphate remobilization from source leaves to roots and younger leaves, was identified in response to both severe and medium P‐deficiency, with a stronger increase under severe P‐deficiency during tuberization (Figure 3A). *Phosphate remobilization from older leaves to younger leaves entails phosphate export into the apoplast by PHO1 followed by the loading of phosphate through PHT1;5 into the phloem (*Stefanovic et al., 2011). Expression of *PHO1* genes in leaves was significantly increased under both medium and severe P‐deficiency conditions at both developmental stages, with stronger induction observed under severe deficiency during tuberization (week 6) (Figure 3A). In the context of PHT1;7, a study on *StPHT1;7* overexpression in potato suggested that it enhances P use efficiency (PUE), thereby promoting potato growth and development (Cao et al., 2020). Expression of *StPHO2* (*Soltu.DM.02G017630*), which encodes the ubiquitin‐conjugating enzyme UBC24 that promotes degradation of PHT1 and PHO1 (Liu et al., 2012), was downregulated in roots under severe and medium P‐deficiency during tuberization, as well as in leaves under severe P‐deficiency during pre‐tuberization and medium P‐deficiency during tuberization (Figure 3A). Additionally, three genes encoding PHOSPHATE TRANSPORTER TRAFFIC FACILITATOR1 (PHF1), which facilitates the intracellular trafficking of PHT1s from the ER to the plasma membrane (Chen et al., 2011; González et al., 2005), were upregulated in roots and leaves under severe P‐deficiency at both developmental stages, while one gene was upregulated under moderate P‐deficiency during tuberization (Figure 3A). Sulfate transporter 3;4 (SULTR3;4), which belongs to SULTR family and acts as a sulfate transporter‐like P distribution transporter (SPDT), mediates the xylem‐to‐phloem transport of phosphate rather than sulfate, and consequently is involved in the source‐to‐sink allocation of phosphate (Ding et al., 2020). Severe and medium P‐deficiency induced the expression of *StSULTR3;4* (*Soltu.DM.12G010700*) in both roots and leaves at both developmental stages (Figure 3A), indicating a sustained upregulation of this gene in response to fluctuating P availability. Collectively, the transcriptional dynamics of *PHT1s*, *PHF1s*, and *PHO1s* genes vary based on the specific organ, intensity of P‐stress, or developmental stage. Overall, severe P‐deficiency triggered earlier and stronger transcriptional activation of key PHT genes involved in phosphate uptake, translocation, and remobilization. For example, three *StPHT1;3* homologs in the root were upregulated by 2.7 to 3.3‐fold at weeks 3 and 6 under severe P‐deficiency. In contrast, under medium P‐deficiency, the same genes showed weaker induction, with increases of only 1.1 to 1.4‐fold at week 3 and 2.4 to 3.0‐fold at week 6. Similarly, *StPHT1;4* showed 1.6‐fold upregulation at week 3 and 1.0‐fold at week 6 under severe P‐deficiency but remained unchanged under medium P‐deficiency. These results demonstrate that severe P‐deficiency induces a more robust and earlier transcriptional response compared to medium P‐deficiency.

Several studies have reported that under P starvation, plants exhibit an increased capacity for P‐uptake while concurrently decreasing their uptake capacity for NO_3_^−^ (Gniazdowska & Rychter, 2000; Wang et al., 2020). This is likely a homeostatic response to the disruption of N and P stoichiometric ratios by P fluctuations (Güsewell, 2004). Severe P‐deficiency markedly downregulated root‐specific *NRT2.1*, *NRT2.4*, and *NRT2.5* expression, key transporters for high‐affinity NO_3_^−^ uptake under N‐starvation (Lezhneva et al., 2014; O'Brien et al., 2016) (Figure 3A). This repression was strongest at week 6, aligning with greater growth inhibition, lower N‐demand, and an altered C:N ratio. In contrast, moderate P‐deficiency caused milder, stage‐specific downregulation, consistent with less severe growth reduction (Figure 3A).

#### Remodeling of membrane lipids and Pi remobilization‐related transcripts

P‐deficiency triggers lipid remodeling by degrading phospholipids in plastidial and extraplastidial membranes, releasing phosphate for essential functions and providing precursors for non‐P‐containing lipids like galactolipids and sulfolipids, which help to restructure membranes under low‐P conditions (Verma et al., 2021). Under both medium and severe P‐deficiency, genes encoding phospholipid‐degrading enzymes (phospholipase A and phospholipase D) and those involved in diacylglycerol synthesis (glycerophosphodiester phosphodiesterases, glyceraldehyde‐3‐phosphate acyltransferases, 1‐acylglyserol‐3‐phosphate O‐acyltransferases) were upregulated in leaves and roots during pre‐tuberization and tuberization stages (Figure 3B), supporting non‐P lipid formation (Reszczyńska & Hanaka, 2020). Additionally, genes encoding monogalactosyldiacylglycerol (MGDG) synthase and digalactosyldiacylglycerol (DGDG) synthase, which are involved in galactolipid synthesis, as well as genes encoding UDP‐sulfoquinovose synthase (SQD1) and sulfoquinovosyl‐diacylglycerol synthase (SQD2), which are engaged in sulfolipid synthesis (Zhu et al., 2022), were upregulated in the leaves and roots of potato plants under medium and severe P‐deficiency (Figure 3B). Overall, more genes involved in phospholipid degradation and non‐P‐containing lipid biosynthesis were upregulated in leaves than in roots under severe (32 vs. 24) and medium (26 vs. 20) P‐deficiency. Under severe P‐deficiency, the number of upregulated genes was similar between 3‐ and 6‐week‐old plants in both leaves (16 vs. 15) and roots (12 vs. 12). In contrast, under medium deficiency, more genes were upregulated in roots at 6 weeks than at 3 weeks (13 vs. 7), while the opposite trend was observed in leaves (10 vs. 15) (Figure 3B).

We also detected upregulation of numerous acid phosphatases (APases)‐encoding genes under severe P‐deficiency (20 and 21 genes in roots, 19 and 21 in leaves at weeks 3 and 6, respectively) and medium P‐deficiency (16 and 21 in roots, 15 and 20 in leaves at weeks 3 and 6, respectively) (Figure 3C). Increased transcript abundance of these genes involved in recycling phosphate from intra‐ and extracellular P metabolites (Deng et al., 2020; Tran, Hurley, & Plaxton, 2010) and releasing phosphate from organophosphate compounds in soil (Wang et al., 2011) coincided with decreased levels of internal free phosphate in the roots and leaves subjected to severe and medium P‐deficiency (Figure 1A,B). Most P starvation‐inducible APases are purple acid phosphatases (PAPs) that hydrolyze phosphate‐monoesters and phosphoanhydrides (Wang & Liu, 2018). Several *StPAP* genes (*StPAP8*, *StPAP10*, *StPAP12*, *StPAP15*, *StPAP16*, *StPAP17*, *StPAP23*, *StPAP26*, and *StPAP28*) were upregulated in roots and leaves under severe and medium P‐deficiency at weeks 3 and 6, with stronger induction under severe P‐deficiency (Figure 3C), likely reflecting greater internal free phosphate depletion (Figure 1A,B). Consistent with this finding, numerous previous studies have reported the increased transcript abundance of the PAP gene family members in Arabidopsis, such as *AtPAP10* (Wang et al., 2011; Zhang, Wang, et al., 2014), *AtPAP12*, *AtPAP26* (Robinson et al., 2012; Tran, Qian, et al., 2010), *AtPAP15* (Wang, Wang, et al., 2009), *AtPAP17* (O'Gallagher et al., 2022), and *AtPAP25* (Del Vecchio et al., 2014). In response to P starvation, phosphate remobilization takes place through the degradation of nucleic acids (RNA and DNA) by enzymes such as ribonucleases (RNases) and endonucleases (ENDOs) (Gho et al., 2020; Yoshitake & Yoshimoto, 2022). Genes encoding the RNase T2 family, which play a role in phosphate recycling through RNA decay, were upregulated under severe P‐deficiency (8 and 2 genes at week 3 and, 3 and 7 genes at week 6 in the root and leaves, respectively) and medium P‐deficiency (5 and 2 genes at week 3 and, 4 and 7 genes at week 6 in the root and leaves, respectively) (Figure 3C). These findings indicated that upregulation of these genes occurred earlier in the leaves than in the roots during severe and medium P‐deficiency.

In conclusion, transcriptional reprogramming of phosphate transporters, lipid remodeling enzymes, and phosphate‐recycling genes constitutes a tightly coordinated adaptive strategy in potato plants, with responses varying in intensity and timing depending on the severity of P deficiency and developmental stage.

### Sustained accumulation of amino acids during developmental stages under P deficiency: Balancing C–N and storing N for future use

**Figure 4:**
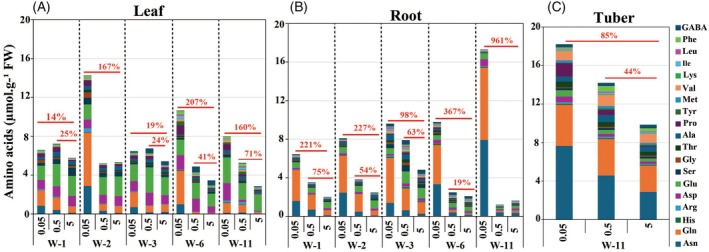
Changes in amino acid levels in leaf (A), root (B), and tuber (C) of potato plants grown under varying P concentrations (0.05, 0.5, and 5 mM KH₂PO₄) across developmental stages (1, 2, 3, 6, and 11 weeks). Data represent the means of four biological replicates. W, week.

The contents of 19 free AAs in potato leaves and roots were measured over the different time points (1, 2, 3, 6, and 11 weeks) in response to fluctuations in P availability (Figure 4; Table S6). The total amount of AAs in leaves changed in response to severe and medium P‐stress across different developmental stages, with the highest accumulation under severe P‐stress (14, 167, 19, 208, and 160% at 1, 2, 3, 6, and 11 weeks, respectively) (Figure 4A; Table S6a). Earlier studies on Arabidopsis (Chutia et al., 2021; Pant et al., 2015), barley (Alexova et al., 2015), soybean (Li et al., 2023), and wheat (Zheng et al., 2021) also found an accumulation of AAs under P‐limiting conditions, while a study on maize reported decreased levels of AAs under P‐deprivation (Luo et al., 2019). In roots, AAs accumulated even to a greater extent than in leaves, which was seen already after 1 week of P‐stress, and their accumulation was intensified during prolonged P‐stress (Figure 4B; Table S6b). Thus, the total contents of AAs in the roots at 1, 2, 3, 6, and 11 weeks increased by 22, 227, 99, 366, and 961%, respectively, under severe P‐stress and by 75, 55, 63, 19, and 25%, respectively, under medium P‐stress (Figure 4B; Table S6b). In conclusion, potato plants (leaves and roots) prioritized AA accumulation over the ATP‐intensive process of protein synthesis during P‐stress (Figure S4). This suggests that storing AAs might be a preparatory mechanism to enable rapid protein synthesis once sufficient P is available, reflecting a resource‐conservative strategy. In tubers, AA content increased by 85% and 44% under severe and medium P‐stress, respectively (Figure 4C; Table S6c). The relatively lower increase of AAs in tubers under P‐stress compared to roots and leaves suggests that the storage capacity of AAs in tubers, as a major N‐sink, may be constrained by an upper limit (beyond which further accumulation does not occur even under P‐stress). Which AAs and to what extent they accumulate were influenced by the severity of P‐stress.

Among the enriched AAs, Asn and Gln, both characterized by a high N:C ratio, showed the most pronounced and progressive increase throughout all developmental stages and organs, with the highest accumulation observed under severe P‐deficiency (Table S6). The continuous accumulation of Asn and Gln under severe P‐deficiency suggests a sustained adaptation strategy, whereby plants enhance N storage and redistribution to maintain metabolic balance and support survival under limited P availability. In addition to Gln and Asn, elevated levels of other AAs with a high N:C ratio (Arg, His, and Lys) were detected in the roots and leaves, especially under severe P stress (Table S6a,b). González‐Hernández et al. (2019) reported a striking increase in Gln, Arg, Asn, His, and Lys levels in the leaves of NH_4_^+^‐supplied tomato plants. At least two possible explanations exist for the accumulation of N‐rich AAs under P limitation. One possibility is that N supply exceeds demand under P‐stress, but C skeletons are limiting due to reduced photosynthetic C fixation (Huang et al., 2008). Consequently, the precise regulation of the C‐N balance becomes crucial. The robust accumulation of N‐rich AAs, which integrate 2–4 NH_4_^+^ molecules into a single C skeleton, serves as a valuable mechanism for effectively managing the C‐N balance. The second possibility may involve sustained NH_4_^+^ uptake by the roots under P stress, which enhances rhizosphere acidification and thereby increases P solubility and uptake (Tian et al., 2021), or the activation of metabolic processes contributing to NH₄^+^ release (such as AA catabolism, increased activity of phenylalanine ammonia‐lyase and glutamate dehydrogenase, and photorespiration) (Li et al., 2007; Usadel et al., 2008), which requires rapid NH_4_^+^ assimilation. RNA‐seq data indicated that the expression levels of *AMMONIUM TRANSPORTER 1;1* (*AMT1;1*) and *AMT1;2* did not exhibit a decreased expression in the roots under P stress, despite reduced plant growth and consequently lower NH₄^+^ demand. Likewise, transcript levels of high‐affinity NRTs, such as the NTR2‐related genes (*NRT2;1*, *NRT2;4*, and *NRT2;5*), were reduced in the roots under P stress (Figure 3A). Therefore, we suggest that the increased accumulation of nitrogen‐rich AAs in roots and leaves under low‐P conditions represents a strategy to detoxify excess NH₄^+^, arising from its elevated availability relative to carbon skeletons due to P deficiency‐induced carbohydrate limitation (Huang et al., 2008; Pant et al., 2015).

GABA is a key intermediate produced in the GABA shunt, a pathway that bypasses two steps of the TCA cycle and enables plants to regulate C and N metabolism under stressful conditions (Benidickson et al., 2023). The GABA shunt can be activated under stress conditions that deplete thiamine diphosphate, an essential cofactor for 2‐oxoglutarate dehydrogenase (Joshi et al., 2019). In response to medium and severe P‐deficiency, we observed a transient increase in GABA content in leaves (37% and 113%, respectively) and roots (84% and 60%, respectively) during the first week. Additionally, under severe P‐deficiency, GABA levels in roots increased by 39% in the second week but showed no significant changes at later time points (weeks 3, 6 and 11) (Figure S5; Table S6a,b). This may suggest that GABA accumulation is part of an early, but transient metabolic response to P‐deficiency, rather than a prolonged adaptation strategy to facilitate organic acid synthesis by bypassing two reactions of the TCA cycle.

In the tubers, proline exhibited the highest increase among the AAs detected under severe and medium P‐stress (11.9‐ and 4.4‐fold, respectively) (Figure 4C; Table S6c). Proline is a critical molecule in stress tolerance, contributing to osmotic regulation, stabilization of cellular structures, detoxifying reactive oxygen species (ROS), and maintaining redox balance under unfavorable environmental conditions (Aleksza et al., 2017). Proline accumulation reflects the tuber's adaptive strategy to preserve metabolic balance without significantly diverting C and N away from starch synthesis. Additionally, proline acts as a compatible solute that supports cellular hydration (Hayat et al., 2012), allowing tubers to endure prolonged P‐stress without compromising their storage function. Its accumulation may also serve as a reservoir for rapid N remobilization during periods of recovery, reinforcing tuber resilience under fluctuating P availability.

Together, the sustained accumulation of free AAs, especially N‐rich ones like Asn, Gln, Arg, His, and Lys, in leaves and roots under medium and severe P‐deficiency, with markedly higher levels under severe P‐stress, reflects a resource‐conservative strategy to balance C‐N metabolism and enhance stress adaptation. This response is complemented by an early and transient accumulation of GABA in roots and leaves, while proline accumulation predominates in tubers.

### Dynamic carbohydrate responses to varying P levels during developmental stages: Growth, storage, and metabolic adjustments

Carbohydrate dynamics in potato plants revealed distinct time‐dependent metabolic strategies in response to medium and severe P‐deficiency.

#### Fine‐tuned C metabolism during weeks 1 to 3 (pre‐tuberization stage) revealed distinct root and leaf responses to P‐stress in potato

**Figure 5:**
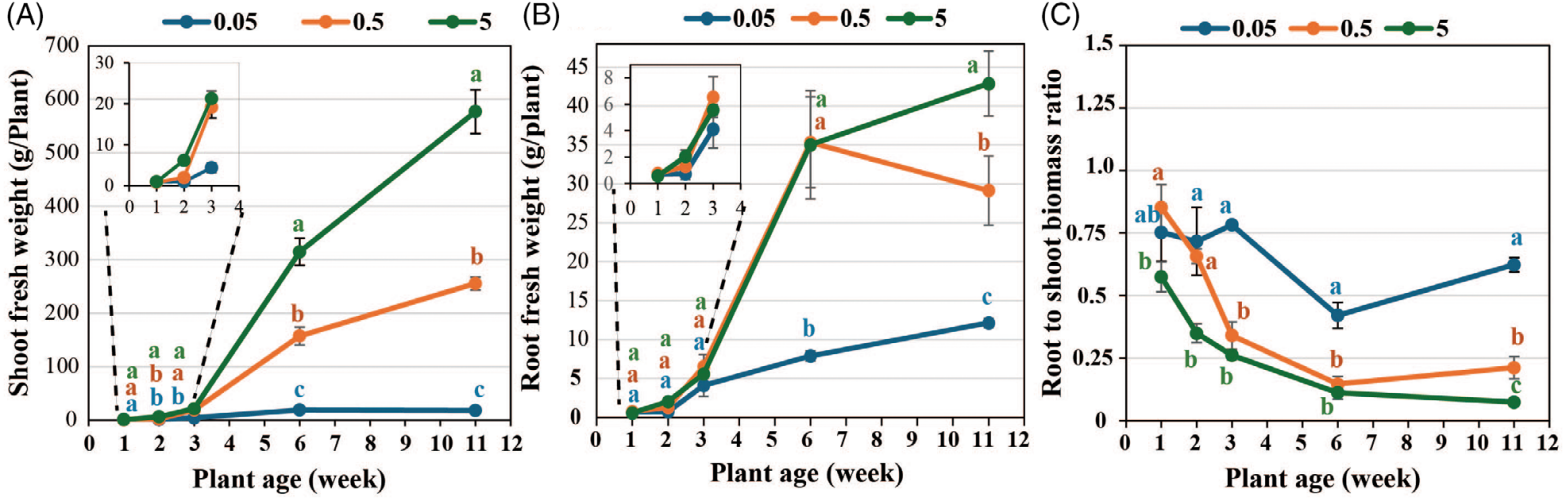
The effects of variation in P‐supply on shoot fresh weight (A), root fresh weight (B), and root‐to‐shoot biomass ratio (C) of potato (*Solanum tuberosum*). The plants were grown on 0.05 (severe P‐deficiency), 0.5 (medium P‐deficiency), or 5 mM P (sufficient P‐availability) supplied as KH_2_PO_4_ for 1, 2, 3, 6, and 11 weeks. Statistical analysis of data was performed at each developmental stage separately. Values represent means±SD of five biological replicates. Different letters indicate statistically significant differences based on Duncan's multiple range test (*P* ≤ 0.05).

**Figure 6:**
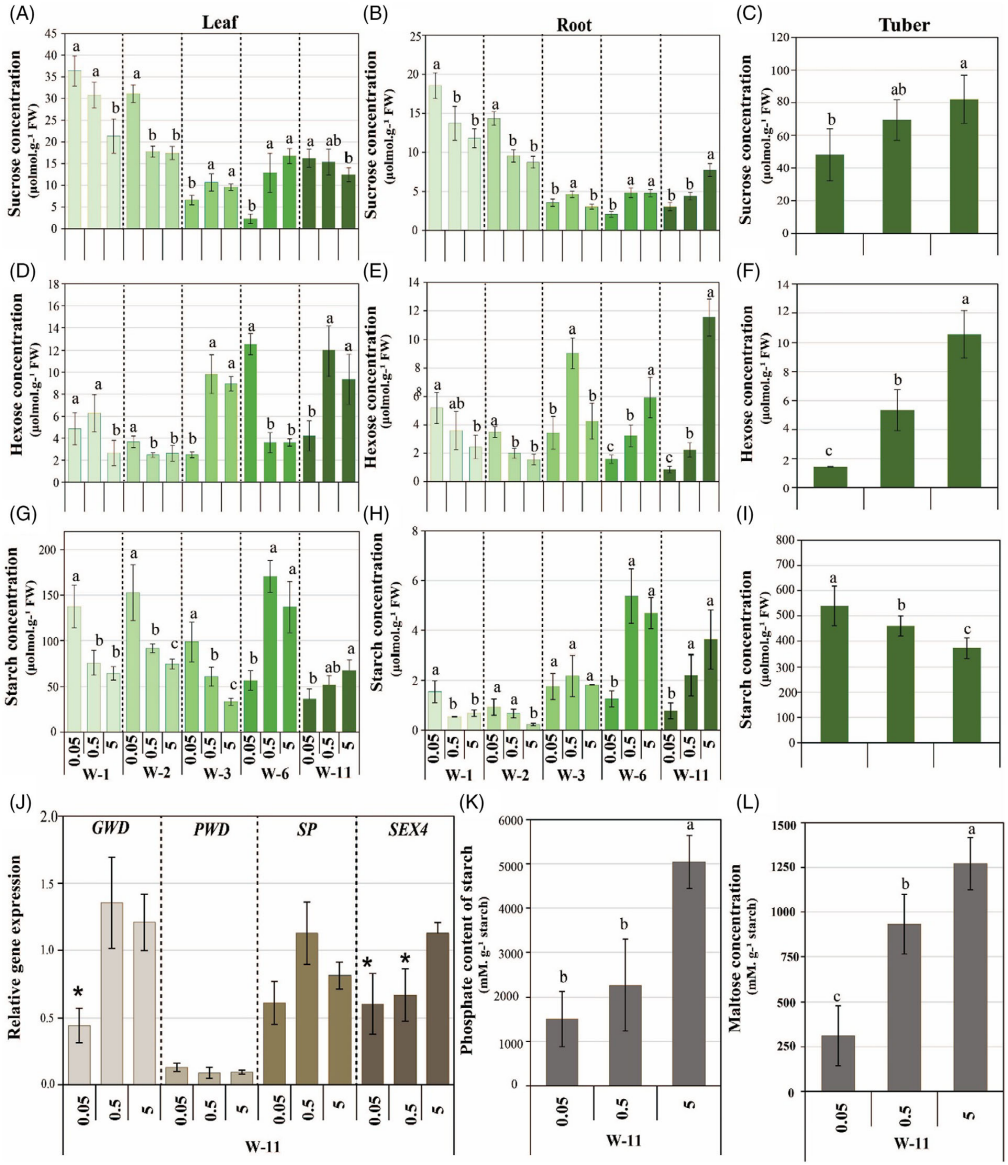
Effects of severe P‐deficiency (0.05 mM) and medium P‐deficiency (0.5 mM), compared with sufficient P‐availability (5 mM), on sucrose (A–C), hexose (D–F), and starch (G–I) levels in leaves, roots, and tubers over time (1, 2, 3, 6, and 11 weeks). Changes in expression levels of starch metabolism‐related genes: *Glucan, Water Dikinase* (*GWD*), *Phosphoglucan, Water Dikinase* (*PWD*), *Starch Phosphorylase* (*SP*), *Starch Excess4* (*SEX4*) (J), phosphate content in starch (K), and maltose content in tubers (L) in tubers under severe and medium P‐deficiency. Values represent means±SD of five biological replicates. Statistical analysis of data was performed at each developmental stage separately. Asterisks indicate significant differences as determined by Student's *t*‐test. W, week.

Despite early sensing of P‐stress via reduced internal phosphate levels at week 1 (Figure 1A,B), no visible decline in shoot and root growth was observed under medium and severe P‐deficiency at this early stage (Figure 5A,B). By week 2, shoot growth declined under severe and medium P‐deficiency (84% and 67%, respectively), while root growth remained comparable to sufficient P‐supplied plants (Figure 5A,B; Figure S6). Therefore, P‐stress first impaired aboveground growth, while root development remained unaffected for optimal phosphate foraging. Correspondingly, the root‐to‐shoot biomass ratio increased under medium P‐deficiency at week 1 and under both P‐stress levels by week 2 (Figure 5C). Under medium P‐deficiency, leaf‐sucrose and leaf‐hexose contents increased by 44% and 203%, respectively, at week 1 (Figure 6A,D), while leaf starch (Figure 6G) and all root carbohydrates remained unchanged (Figure 6B,E). However, by week 2, starch levels increased by 68% in leaves and 190% in roots (Figure 6G,H), with no further changes in sucrose and hexose compared to sufficient P in both organs (Figure 6A,B,D,E). These findings suggest that medium P‐stress elicits an early accumulation of soluble sugars in leaves at week 1, followed by enhanced starch accumulation in both leaves and roots by week 2, reflecting a coordinated metabolic adaptation to buffer energy demands under medium P‐stress. In contrast, severe P‐deficient plants showed significantly elevated concentrations of hexose, sucrose, and starch in both leaves and roots as early as week 1 (in leaves: hexose by 135% and 40%, sucrose by 70% and 78%, and starch by 112% and 320%; in roots: hexose by 113% and 125%, sucrose by 57% and 64%, and starch by 125% and 330% at week 1 and week 2, respectively) (Figure 6A,B,D,E,G,H). The stronger and earlier accumulation of sugars and starch under severe P‐deficiency, compared to medium P‐stress, in both leaves and roots (Figure 6A,B,D,E,G,H) reflects a more pronounced and rapid adaptive response, wherein the plant prioritizes energy storage and C conservation, most likely in anticipation of prolonged P‐limitation. These findings showed that despite the reduction in internal free phosphate in leaves during weeks 1 and 2 (Figure 1A), photosynthetic activity might persist to some extent, allowing ongoing sucrose synthesis, consistent with previous reports showing only minor changes in photosynthetic rates during the early stages of P‐deficiency (Ciereszko et al., 1996; Xiao et al., 2024). Furthermore, P‐starvation‐induced sucrose accumulation initiates sugar signaling cascades leading to alterations in the expression of some P starvation‐responsive genes (Hammond & White, 2011; Rouached et al., 2010). High root‐sucrose levels in severe P‐deficient plants at weeks 1 and 2 may result from increased sucrose translocation to the root (Hammond & White, 2008) and/or reduced sucrose hydrolysis (Sun et al., 2024; Xiao et al., 2024).

**Figure 7:**
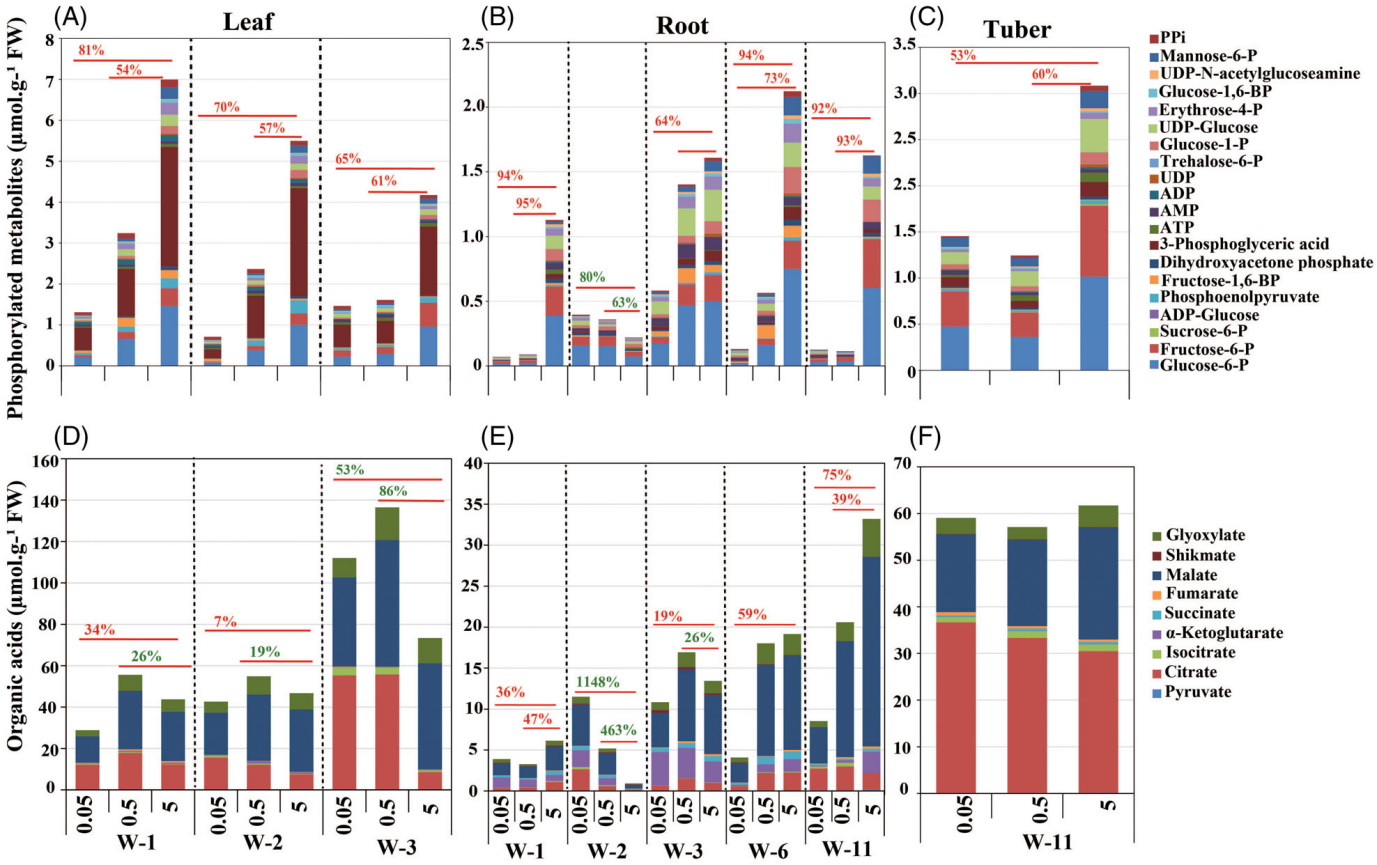
Effect of severe P‐deficiency (0.05 mM) and medium P‐deficiency (0.5 mM), compared with sufficient P‐availability (5 mM), on the levels of phosphate metabolites (A–C) and organic acids (D–F) in leaf, root, and tuber of the potato plants during different developmental stages. W, week.

Although root hexose levels increased under severe P‐deficiency, most likely due to sucrose hydrolysis (Figure 6E), the concurrent decrease in hexose‐phosphates and other key glycolytic intermediates (fructose‐1,6‐bisphosphate, dihydroxyacetonphosphate (DHAP), 3‐phosphoglyceric acid (3PG), phosphoenolpyruvate (PEP), and pyruvate) in roots during week 1 (Figure 7B; Figure S7; Table S7) suggests a downregulation of energy‐intensive processes to conserve P for ATP synthesis and metabolic regulation. This early metabolic adjustment in already one‐week‐old roots might reflect an adaptive response rather than a metabolic failure. By suppressing energy‐demanding pathways while maintaining a basal level of carbon metabolism, P‐deficient plants conserve P resources and prevent premature growth inhibition at week 1.

Likewise, the reduction in hexose‐phosphates and other glycolytic intermediates in medium P‐deficient roots (Figure 7B; Figure S7; Table S7), despite unchanged sucrose and hexose levels at this stage (Figure 6B,E), suggests a partial downregulation of glycolysis as an early adaptation to conserve P under moderate P‐stress. Additionally, the decrease in TCA cycle‐related organic acids (citrate, isocitrate, succinate, fumarate, and malate) in medium and severe P‐deficient roots at week 1 (Figure 7B; Figure S7; Table S7) likely reflects a combination of reduced synthesis and enhanced exudation to mobilize phosphate from the soil. Interestingly, the approximately similar reduction extent of hexose‐phosphates, glycolytic intermediates, and TCA cycle‐related organic acids under both P‐stress conditions at week 1 might suggest conservation of internal P as a general early strategy regardless of the severity of P‐deficiency (Figure 7; Figure S9; Table S7). Unlike in week 1, hexose‐phosphates and glycolytic intermediates (fructose‐1,6‐bisphosphate, 3PG, DHAP, and PEP) increased in the roots under medium and severe P‐deficient conditions at the subsequent time point (week 2), suggesting an attempt to restore glycolytic flux. Increased levels of organic acids were observed in the roots in response to medium and severe P‐deficiency (Figure 7B; Figure S8A,B; Table S7), which may support further exudation to facilitate P‐acquisition by mobilizing external phosphorus sources (Vance et al., 2003).

By week 3, medium P‐deficiency had no significant effect on shoot and root growth or root‐to‐shoot biomass ratio (Figure 5A–C), indicating a transient decline in shoot growth at week 2 with subsequent recovery. In contrast, severe P‐deficiency reduced shoot growth by 79%, while root growth remained comparable to sufficiently P‐supplied plants, indicating sustained investment in root growth for improved P‐acquisition, resulting in a high root‐to‐shoot biomass ratio (Figure 5A–C). Medium P‐deficiency increased leaf starch by 82% without altering leaf sucrose or hexose contents (Figure 6A,D,G), and raised root sucrose and hexose by 52% and 112%, respectively, with no change in root starch (Figure 6B,E,G). In contrast, severe P‐deficiency markedly reduced leaf‐sucrose (31%) and leaf‐hexose (72%) but increased leaf starch by 195% (Figure 6A,D,G), while root carbohydrates remained unaffected (Figure 6B,E). Thus, during the vegetative stage, leaf‐starch accumulation under medium P‐deficiency was gradual and sustained, whereas under severe P‐deficiency, it was strong and rapid, but transient. This suggests that medium P‐deficient plants engage in a more balanced metabolic adjustment to cope with limited P availability. In contrast, the lower starch accumulation in 3‐week‐old leaves, compared to 2‐week‐old leaves, under severe P‐deficiency (195% vs. 330%) (Figure 6G) may reflect limited carbon availability or metabolic constraints caused by prolonged severe P‐stress.

In medium P‐deficient 3‐week‐old roots, sucrose and hexose accumulated (Figure 6B,E), but hexose‐P levels remained unchanged (Figure S7), suggesting that sucrose translocation and hydrolysis may outpace hexose phosphorylation, likely due to limited P availability in the root. Unchanged root hexose‐phosphate levels under medium P stress, along with elevated root fructose‐1,6‐bisphosphate and reduced concentrations of 3PG and PEP (Figure S7), indicate a downregulation of glycolytic flux downstream of fructose‐1,6‐bisphosphate. Pyruvate levels were stable, possibly due to reduced consumption rather than increased production. These findings suggest that under moderate P deficiency, glycolytic flux in the roots is moderately reduced, likely as a P‐saving response. However, the extent of these reductions appears finely tuned, as it does not impair the production of TCA cycle‐related organic acids, which remain comparable to sufficiently P supplied roots (Figures S7 and S8; Table S7), indicating a maintained capacity for energy metabolism and carbon flow under moderate P stress. In contrast, severe P deficiency led to reductions in hexose‐phosphates (Glucose‐6P, Fructose‐6P) and key glycolytic intermediates (DHAP, 3PG, PEP) in the roots, together with the decreased levels of root TCA cycle‐related organic acids such as citrate, isocitrate, malate, and fumarate (Figures S7 and S8; Table S7), indicating a substantial slowdown of central carbon metabolism. This likely reflects a strong P‐saving response, where both glycolytic and mitochondrial fluxes are downregulated to limit ATP and P consumption, ultimately compromising biosynthetic and energetic outputs in the root (Roychowdhury et al., 2023). Additionally, greater reductions of phosphorylated metabolites in leaves than in roots under both P‐deficient conditions at week 3 (Figure 7A,B) may reflect a strategic P allocation to sustain root metabolism and growth. Since roots are crucial for P foraging, plants may prioritize C flux and energy supply of roots at the expense of leaf metabolism. This shift suggests a reprogramming of carbon metabolism under P stress, where reduced photosynthetic carbon input in leaves is redirected to maintain metabolic activity in roots to support P acquisition and adaptive growth responses.

In conclusion, over the first three weeks of P‐deficiency, potato plants fine‐tune carbon metabolism in an organ‐ and stress‐level‐specific manner, initially prioritizing root growth and sugar accumulation, followed by sustained starch buildup in leaves and adaptive metabolic downregulation in roots. A notable metabolic peak at week 2 suggests a critical adjustment phase, especially under severe P‐stress. Medium P‐deficiency led to a balanced and recoverable adjustment, while severe P‐deficiency triggered rapid but metabolically costly responses, compromising shoot growth and carbon availability by week 3.

#### Differential impacts of medium and severe P‐deficiency on C allocation and metabolic regulation during the potato tuber development

During tuberization (week 6), shoot growth declined by 94% and 50% under severe and medium P‐deficiency, respectively. However, while root growth was significantly reduced under severe P‐deficiency (by 77%), it remained unchanged under medium P‐deficiency, likely to sustain P‐acquisition (Figure 5A,B; Figure S6). At this developmental stage, plants enter the tuberization phase, where developing tubers act as strong carbon sinks, demanding high sucrose levels for starch synthesis (Gong et al., 2023; Zrenner et al., 1995). However, under severe P‐deficiency, a significant decline in photosynthesis (CO₂ assimilation rate) at this stage (Figure S9) resulted in an 86% reduction in leaf sucrose (Figure 6A), impairing the plant's ability to meet this demand. To compensate, severe P‐deficient potato plants may remobilize leaf starch, as supported by a 58% reduction in leaf starch levels (Figure 6G). This breakdown of leaf starch might provide an alternative carbon source to support sucrose export from the leaves to developing tubers under P stress. Additionally, the decline in leaf starch under severe P‐deficiency likely resulted from a reduction in starch synthesis due to decreased photosynthesis. Sucrose and starch levels also decreased in the roots (56% and 73%, respectively) (Figure 6B,H), indicating that under severe P‐deficiency, roots received less sucrose as the plant redirected carbon allocation toward developing tubers. This adjustment ensures that carbon resources are prioritized for tuber growth, which is crucial for survival and future reproduction under P‐limited conditions. In contrast, medium P‐deficiency did not lead to significant changes in sugar or starch levels in leaves and roots (Figure 6), further supporting the idea that plants under moderate P stress maintained a more balanced metabolic state to avoid drastic reallocation or depletion of carbon reserves. Correspondingly, metabolic profiling in the roots revealed a strong depletion of glycolytic intermediates under severe P‐deficiency, compared to medium P‐deficiency (Figure 7B,E; Figure S7; Table S9). Under medium P‐deficiency, glycolytic intermediates in the roots decreased similarly to those under severe P‐deficiency conditions, but to a lesser extent, aligning with unaltered sucrose and starch levels in roots (Figure 6B,H; Figure S7). These results suggest that under medium P‐deficiency, 6‐week‐old plants can sustain central carbon metabolism and prioritize balanced and stable root growth and carbon allocation, while severe P‐deficiency disrupts primary metabolism and induces stronger shifts in resource partitioning toward tuber development at the expense of root maintenance (Figure 5B). Additionally, under both medium and severe P‐deficiency, glycolytic intermediates and TCA cycle‐related organic acids decreased more strongly in roots than in leaves at week 6 (Figure 7A,B,D,E; Figures S7 and S8; Table S7), indicating a pronounced disruption of carbon metabolism in root organs. Collectively, these findings suggest that during tuber development, prolonged severe P stress drives a marked shift in carbohydrate partitioning toward reproductive sinks at the expense of vegetative organs, whereas under medium P stress, metabolic adjustments remain moderate, supporting both vegetative growth and tuber development.

Medium and severe P‐deficiency also elicit distinct impacts on carbon allocation and metabolism during the tuber‐filling stage. By week 11, severe P‐deficient plants showed a 30% increase in leaf‐sucrose levels, accompanied by declines of 54% in leaf‐hexoses and 46% in leaf starch (Figure 6A,D,G). This pattern likely reflects the accumulation of unmetabolized sucrose due to reduced carbon utilization in the leaves, driven by a 97% reduction in shoot growth (Figure 5A) and, consequently, diminished sucrose demand. Concurrently, the pronounced decreases in root sucrose (60%) and tuber sucrose (41%) under severe P deficiency (Figure 6B,C) suggest impaired translocation, likely due to limited energy availability, leading to sucrose retention in the leaves. Efficient sucrose transport and utilization are essential for tuber formation and filling (Ševčíková et al., 2017); however, P‐deficiency likely weakens sink strength (Pieters et al., 2001), thereby reducing sucrose uptake and storage in the tubers. Consequently, tuber filling is slowed, leading to fewer and smaller tubers under severe P‐deficiency (Figure 1D,F). Additionally, these severe P‐deficient plants showed a 72% reduction in root growth (Figure 5B), and the strong reduction in root hexose (93%) (Figure 6E) further indicates a severe disruption of sink activity. These changes were accompanied by a much stronger decline in phosphorylated metabolites and TCA cycle‐related organic acids in severe P‐deficient roots than in severe P‐deficient tubers (Figure 7B,C,E,F; Figures S7 and S8; Table S7), indicating that although tuber metabolism was negatively affected by P‐deficiency, it remained more active than in roots. This may reflect a strategic allocation of resources to maintain storage organ function.

Notably, starch content in severely P‐deficient tubers increased by 44% (Figure 6I), likely due to reduced degradation rather than enhanced synthesis. Since starch breakdown requires prior phosphorylation by glucan water dikinase (GWD), phosphoglucan water dikinase (PWD), and subsequent dephosphorylation by SEX4 (Helle et al., 2018; Ritte et al., 2004), the observed 70% reduction in P‐bound starch (Figure 6K) along with the down‐regulation of *GWD* and *SEX4* gene expression (Figure 6J) in tubers of severe P‐deficient plants suggests impaired starch degradation. Additionally, a 71% decline in maltose levels (Figure 6L) further supports reduced starch hydrolysis, indicating a metabolic shift toward carbon conservation and storage under severe P‐limitation.

Under medium P‐deficiency for 11 weeks, shoot growth was reduced by 50% (Figure 5A), while leaf‐sucrose and leaf‐hexose levels remained stable (Figure 6A,D), suggesting that sugar production and hydrolysis exceeded the reduced demand from the shoot. Moreover, although tuber‐sucrose and tuber‐hexose concentrations were maintained (Figure 6C,F), their root levels declined by 43% and 81%, respectively (Figure 6B,E), indicating a possible shift in carbon allocation or a reduction in root sink strength under P‐limited conditions. In parallel, the levels of hexose‐phosphates, other glycolytic intermediates (fructose‐1,6‐bisphosphate, DHAP, 3PG, PEP, and pyruvate), and TCA cycle‐related organic acids (malate, α‐ketoglutarate, succinate, and fumarate) decreased in all organs, with the strongest reductions observed in the roots (Figures S7 and S8; Table S7). These results suggest that sucrose translocation from source leaves to tubers remained active, and tuber metabolism continued, whereas carbon flow to the roots diminished, coinciding with reduced root metabolic activity. This interpretation is supported by the observed 32% reduction in root biomass (Figure 5B) and the stable fresh weight of individual tubers (Figure 1F), which came at the expense of a 55% reduction in tuber number (Figure 1D). In medium P‐deficient tubers, the 33% increase in starch (Figure 6I), along with a 27% decrease in maltose and a 55% reduction in P‐bound starch (Figure 6K,L), indicates reduced starch degradation, albeit to a lesser extent than in severely P‐deficient tubers. Overall, the differential temporal and organ‐specific regulation of carbohydrate metabolism underlines the potato plant's capacity to flexibly balance growth, storage, and stress adaptation in response to varying P availability.

## CONCLUSION

This study uncovered that potato plants employ distinct, organ‐ and stage‐specific strategies to adapt to available P fluctuations. Transcriptomic and metabolic analyses showed that leaves initiate early responses at the pre‐tuberization stage, while roots exhibited stronger transcriptional reprogramming during tuberization. Metabolite profiling revealed a preferential accumulation of N‐rich AAs in leaves and roots, an early but transient increase in GABA in both organs, and elevated proline levels in tubers, suggesting coordinated strategies to regulate C‐N balance and support stress adaptation. Carbohydrate analysis highlighted contrasting responses to P availability: under medium P‐deficiency, sugar and starch levels rose moderately, supporting growth and stable metabolism. In contrast, severe P‐deficiency triggered rapid sugar and starch accumulation in leaves and roots, followed by metabolic repression and carbon reserve depletion. This was accompanied by suppressed glycolysis and TCA cycle activity, especially in roots, suggesting a shift toward carbon conservation and prioritization of tuber development under extreme stress. Understanding these time‐ and organ‐specific metabolic shifts offers valuable insight for breeding and agronomic strategies aimed at enhancing resilience to P‐deficiency in potato and other crops. Together, these findings underscore the dynamic and flexible nature of metabolic and transcriptional responses to varying P levels in potato. They highlight how carbon partitioning and energy metabolism are fine‐tuned across organs and developmental stages to optimize survival and productivity. This work provides key insights that can inform future strategies to enhance nutrient use efficiency and improve stress resilience in crops.

## MATERIALS AND METHODS

### Plant growth

Potato (*Solanum tuberosum* L. cv. Solara) seedlings propagated in tissue culture on MS‐medium (Murashige & Skoog, 1962) containing 2% (*w*/*v*) sucrose were transferred to 2.5‐L pots filled with perlite and were further cultivated in a growth chamber with 16‐h light at 20°C and 8‐h darkness at 18°C. The light intensity was approximately 400 μmol quanta m^−2^ s^−1^. Fertilization was carried out three times per week using a modified Hoagland solution (Table S8). Potato plants grown under severe P‐deficiency (0.05 mM KH_2_PO_4_), medium P‐deficiency (0.5 mM KH_2_PO_4_), or sufficient P availability (5 mM KH_2_PO_4_) were harvested at different developmental stages, including vegetative (1,2 and 3‐week‐old plants), tuberization (6‐week‐old plants), and tuber‐filling (11‐week‐old plants). Root, leaf, and tuber organs were harvested at different developmental stages and stored at −80 C for further analysis.

### RNA extraction, library preparation, and RNA‐seq

Total RNA was extracted from 100 mg of frozen leaf and root organs (*n = 4*) from 3‐ and 6‐week‐old plants, following the protocol by Logemann et al. (1987). Sequencing was performed at the Beijing Genomics Institute. Raw reads (FastQ files) were quality‐checked with FastQC v0.11.9 (https://www.bioinformatics.babraham.ac.uk/projects/fastqc/). The reads were trimmed for adapter content and quality via bbduk v39.01 (https://sourceforge.net/projects/bbmap/). Bases with quality scores below 30 were trimmed from both ends, and reads shorter than 35 bp or with an average quality below 30 were discarded. Trimmed reads underwent a second FastQC check before being aligned to the v6.1 reference genome using STAR v2.7.10a (Dobin et al., 2013). Read counts were obtained with FeatureCounts v2.0.3 (Liao et al., 2014), considering only uniquely mapped reads. Counts were normalized and log‐transformed using the DESeq2 package (v1.38.3) in R (Love et al., 2014). Transcripts with a false discovery rate (FDR) < 0.001 were considered differentially expressed genes (DEGs). The raw sequencing reads have been submitted to the NCBI Sequence Read Archive (SRA) under BioProject ID PRJNA1262896, accessible at: https://www.ncbi.nlm.nih.gov/sra/PRJNA1262896.

### Determination of free phosphate, soluble sugars, starch, anthocyanin, and AAs concentration

Free phosphate concentration in the leaves and roots was measured using a commercial colorimetric phosphate assay kit (SKU No: 700004326, Megazyme, Ireland). For the determination of soluble sugars, starch, and AAS, leaf, root, and tuber organs were extracted with 1 mL of 80% ethanol, following the method described by Hastilestari et al. (2018). Starch hydrolysis and soluble sugar contents were measured according to previously described methods (Hajirezaei et al., 2003). Anthocyanin was extracted using an extraction buffer consisting of methanol containing 1% HCl, and its content was quantified by measuring absorbance at 530 nm (Ito et al., 2015). The levels of AAs were analyzed as previously described by Hofmann et al. (2011) following derivatization with the fluorophore 6‐aminoquinolyl‐N‐hydroxysuccinimidyl carbamate (AQC) (Cohen & Michaud, 1993) using a reversed‐phase HPLC system (Dionex Summit) equipped with a fluorescence detector.

### Phosphorylated intermediates and carboxylates measurements

Phosphorylated intermediates and carboxylates from leaf, root, and tuber samples (50 mg) were extracted with perchloric acid and measured using the protocol previously published by Horst et al. (2009) using ion chromatography with an ICS3000 HPLC system (Dionex) and ESI/MS/MS detection using a QTrap 6500+ Triple‐Quadrupole mass spectrometer with a turboV ion source (Sciex) operated in multiple reaction monitoring mode.

### Starch granules ‐bound phosphate content and maltose levels in tubers

Tuber starch was extracted following the protocol of Liu et al. (2021) and the phosphate content of the starch granules was measured using the method described by Li et al. (2024) with the Malachite Green Phosphate Assay Kit (MAK307, Sigma‐Aldrich). The same extraction used for measuring soluble sugars was also used to measure maltose using the Maltose Assay Kit (MAK513, Sigma‐Aldrich).

### RNA isolation from tubers and quantitative PCR

RNA was isolated from tubers using the CTAB method as described by Gambino et al. (2008). Complementary DNA synthesis was performed as previously described (Ferreira et al., 2010) and quantitative real‐time PCR (qPCR) analysis was conducted using gene‐specific primers listed in Table S9.

### Photosynthesis measurement and total protein content

The rate of CO_2_ assimilation was assessed in fully developed source leaves located on the upper middle stem under greenhouse conditions using a LI‐COR 6800 device. Total soluble protein content in leaves, roots, and tubers was estimated using the Bradford reagent (Bradford, 1976).

### Statistical analysis

Data were subjected to one‐way ANOVA, and significant differences between P treatments for each week were evaluated using Duncan's multiple range test (*P* ≤ 0.05). All statistical analyses were performed using SPSS software version 16.0.

## Supporting information


**Figure S1.** Principal component analysis (PCA) of log‐transformed RNA‐seq counts data.
**Figure S2.** Venn diagram of up‐regulated and down‐regulated differentially expressed genes (DEGs) of transcriptional regulators in potato plants grown under severe P‐deficiency (0.05 mM KH_2_PO_4_), medium P‐deficiency (0.5 mM KH_2_PO_4_), or sufficient P‐availability (5 mM KH_2_PO_4_) over periods of 3 and 6 weeks, corresponding to the vegetative and tuberization stages, respectively.
**Figure S3.** Changes in the expression levels of genes encoding enzymes involved in the biosynthesis pathway of anthocyanins (a), and the levels of anthocyanins (b) in the leaves of potato plants grown under severe P‐deficiency (0.05 mM P), medium P‐deficiency (0.5 mM P), or sufficient P‐availability (5 mM P).
**Figure S4.** Effect of severe P‐stress (0.05 mM) and medium P‐stress (0.5 mM), compared with sufficient P‐availability (5 mM), on the levels of total protein in the leaves (a), roots (b), and tubers (c) of potato plants.
**Figure S5.** The effects of variation in P‐supply on the levels of GABA shunt intermediates in the roots of potato (*Solanum tuberosum*). The plants were grown on 0.05 (severe P‐deficiency), 0.5 (medium P‐deficiency), or 5 mM P (sufficient P‐availability) supplied as KH_2_PO_4_ for 1, 2, 3, 6, and 11 weeks.
**Figure S6.** Photographs of 1‐, 2‐, 3‐, and 6‐week‐old potato plants grown on 0.05 (severe P‐deficiency), 0.5 (medium P‐deficiency), or 5 mM P (sufficient P‐availability) supplied as KH_2_PO_4_.
**Figure S7.** The effects of severe P‐stress (0.05 mM P) and medium P‐stress (0.5 mM P), compared with sufficient P‐availability (5 mM P), on the levels of Glucose‐6P, Fructose‐6P, fructose‐1,6‐bisphosphate (Fru1,6BP), dihydroxyacetonphosphate (DHAP), 3‐phosphoglyceric acid (3PG), phosphoenolpyruvate (PEP), pyruvate, and ATP in the roots over time.
**Figure S8.** The effects of severe P‐stress (0.05 mM KH_2_PO_4_) and medium P‐stress (0.5 mM KH_2_PO_4_), compared with sufficient P‐availability (5 mM KH_2_PO_4_), on the levels of TCA cycle‐related organic acids (malate, isocitrate, citrate, α‐ketoglutarate, succinate, and fumarate) in roots over time.
**Figure S9.** The effects of variation in P‐supply on CO_2_ assimilation in potato (*Solanum tuberosum*).

**Table S1.** List of down‐regulated genes in the leaves in response to P‐deficiency.

**Table S2.** List of up‐regulated genes in the leaves in response to P‐deficiency.

**Table S3.** List of down‐regulated genes in the roots in response to P‐deficieny.

**Table S4.** List of up‐regulated genes in the roots in response to P‐deficieny.

**Table S5.** Up‐ and down‐regulated genes encoding transcriptional regulatory elements in response to P‐deficieny.

**Table S6.** Effects of P‐deficiency on the levels of amino acids in leaves, roots and tuber.

**Table S7.** Effects of P‐deficieny on the levels of metabolites in leaves, roots and tubers.
**Table S8.** Macro‐ and micro‐element composition of nutrient solution used for irrigation of potato plants grown under sufficient P‐availability (5 mM P), medium P‐deficiency (0.5 mM P), and severe P‐deficiency conditions (5 mM P).
**Table S9.** List of genes and their specific primers used in qRT‐PCR.

## Data Availability

The data that support the findings of this study are openly available in NCBI Sequence Read Archive (SRA) at https://www.ncbi.nlm.nih.gov/sra/PRJNA1262896, reference number PRJNA1262896.
